# The Book World of Medicine and Science

**Published:** 1901-11-23

**Authors:** 


### THE BOOK WORLD OF MEDICINE AND SCIENCE.

ALCOHOLISM: A STUDY IN HEREDITY. By G. ARCHDALL REID, M.B., C.M., F.R.S.E. (London: T. Fisher Unwin. 1901. Price 6s. net.)

DR. ARCHDALL REID may properly be called a strong writer. He has an easy flow of somewhat forcible language, he sets his ideas before his readers in quite unequivocal terms, and his ideas are fairly crisp and definite. Hence we may opine that such a presentment of them as is here given will draw down upon him much criticism and perhaps a good deal of abuse. Indeed this book is a veritable trailing of the coat in the Donnybrook Fair of teetotal controversy, and we shall be considerably surprised if some of our total abstinence friends do not tread on the tail of it. Its object appears to be, in the first place, to trace the causes of intemperance on scientific lines, and in the second to indicate what its author calls "a practical remedy." As to the first point he draws a wide distinction between drinking and drunkenness. Men drink alcoholic solutions, he says, for three distinct reasons: to satisfy thirst, to gratify taste, and to produce a distinct effect upon the brain; and it is only the drinking which is done for the latter of these which is a cause of drunkenness. Sober people do not keep sober by dint of self-control. Their sobriety is no particular credit to them. If they do not drink it is merely that they are not tempted so to do. Self-control is then a subordinate affair in the causation of sobriety, lack of temptation or desire being the principal factor. What, then, is the cause of that craving for alcohol which leads to drunkenness? Here we come into the midst of the argument, and this is the leading topic of the book. Dr. Archdall Reid says that every drunkard must be so constituted as to be capable of enjoying deep indulgence, whether in the form of positive pleasure or as a means of relieving physical and mental discomfort or pain, and this faculty, this capacity for enjoying drink, "is certainly inborn." Of course there are other factors in the case, such as the man's knowledge of alcohol, his "recollection of the pleasurable sensations which former acts of drunkenness aroused in him," and the fact that "the more a drinker indulges in drink the more, within limits, does he crave for drink." For all that the potential drunkard is born not made, and thus there are two methods of temperance reform open to us—what may be called Nature's method by the elimination of the excessive drinker, and the temperance reformer's method by the elimination of the drink. These methods are plainly antagonistic. If drink be abolished the potential drunkard is preserved; if the potential drunkard is to be eliminated this can only be done by means of the evil effects of drink upon him. The question then is which method ought we to adopt? Which is the more practicable? Which offers the more certain and easy success? To this Dr. Reid answers without hesitation, Nature's method, the elimination of the potential drunkard. His solution of the problem is then that drunkards should be prevented from reproducing their like. "If drunkards were taken before magistrates, sitting in open or secret session as the accused preferred, and, on conviction, were warned that the procreation of children would subject them to this or that penalty, say a month's imprisonment, the birth-rate of drunkards would certainly fall immensely." This, then, is Dr. Archdall Reid's great scheme. In the meantime, as he says, there need be no relaxation of temperance effort, so far as it involves the saving of individual drunkards, provided always that we forbid children to them. But we must not abolish drink. If we did, we could not discover the drunkard! This is, of course, a little drawback, but as "we must in any case have drunkards till no one enjoys being drunk" the drawback is not so serious as one might suppose.

ESSAYS ON CONSUMPTION, TOGETHER WITH SOME CLINICAL OBSERVATIONS AND REMARKS UPON PNEUMONIA. By J. EDWARD SQUIRE, M.D.Lond., D.P.H.Camb. With an introduction by Sir WILLIAM BROADBENT, Bart., M.D., F.R.C.P. (London: The Sanitary Publishing Co. 1900. Price 10s. 6d. net.)

THIS is a very interesting collection of papers and articles read at various times before the Societies, or published in the medical journals. Appearing as they here do in volume form they give a good summary of the etiology and treatment of consumption, although prominence is naturally given to those aspects of the subject upon which Dr. Squire has done special work. Thus we find the question of heredity very fully discussed, the conclusion arrived at being that the children of parents who are in poor health, whether this arise from phthisis or from other cause, are likely to be themselves [illegible]eakly and thus predisposed to contract any disease to t[illegible]e infection of which they may be exposed. In addition to this, however, if the ill-health of the parents has been due to phthisis, their delicate children, being constantly exposed to the infection of tuberculosis, are especially liable to become tuberculous. An interesting fact is shown by the statistics which Dr. Squire has collected, namely that phthisis in the mother is more likely than phthisis in the father to be followed by the same disease in the children; the explanation being that while the father is away from the home a good portion of his time the phthisical mother is at home and infecting her house and family all day long. In the chapter on treatment a survey is given of various methods, and a good deal is said about the hygienic treatment of consumption at home. Altogether these papers form a very interesting volume, and they hang together much better than is often the case with collected essays.

GOLDEN RULES FOR DISEASES OF CHILDREN. By GEORGE CARPENTER, M.D.Lond., M.R.C.P. "Golden Rules," Series, No. xi. (Bristol: John Wright and Co. Price 1s.)

THIS little book is full of good advice. On the principle that all knowledge is good, even when taken in small doses, there is something to be said for these very little booklets. It is obvious, however, that anyone who wishes to know about diseases of children must read a vastly bigger book than this, while if he does read such a book, these "Golden Rules" will hardly be necessary. Still they have a place. To students who, having already mastered the subject, merely wish to run over it, and to practitioners who like to have something to read on their rounds, these "Golden Rules" may be of service, and this one is a very good specimen of the series. Although it does not, and of course cannot, give much about any one disease, it touches upon most of the maladies to which children are liable, and its short snappy sentences can be easily digested on occasions when there is no time for anything which requires continuous thought.

